# Beyond the ethics deflection: Why patient burden is a scientific review question, not just an IRB concern

**DOI:** 10.1186/s40900-026-00927-z

**Published:** 2026-07-06

**Authors:** Srdjan Stakic

**Affiliations:** Stanford Cancer Institute Scientific Review Committee, Stanford Cancer Center Patient and Family Advisory Council, Stanford Medicine Community Advisory Board for Clinical Research, Stanford, CA USA

**Keywords:** Patient and public involvement, Clinical trial design, Scientific review, Institutional review board, Patient burden, Trial feasibility, Research ethics, Co-production

## Abstract

Patient advocates increasingly serve as voting members of Scientific Review Committees (SRCs) charged with evaluating the scientific merit and feasibility of clinical trial protocols. In principle, this gives advocates a seat at the table when the scientific architecture of a trial is most malleable. In practice, a recurring pattern undermines that promise. When advocates raise concerns about visit frequency, procedural invasiveness, or restrictive eligibility criteria, those concerns are often deflected on the grounds that they are ethical rather than scientific, and therefore properly the province of the Institutional Review Board. This Comment names that pattern jurisdictional dismissal and argues that it rests on a category error. Patient burden is a primary determinant of accrual, retention, data completeness, and generalizability. These are scientific concerns by any reasonable definition. When SRCs decline to engage with them, they let an unsuccessful trial proceed to ethics review with a fixed design that the IRB cannot meaningfully alter. I describe how jurisdictional dismissal operates, distinguish the scientific feasibility question that belongs to the SRC from the ethical acceptability question that belongs to the IRB, and propose four concrete changes to SRC practice that recognize feasibility as a scientific criterion in its own right.

## Abstract (plain language summary)

When patients and former research participants join clinical trial review committees, they bring firsthand experience of what it can be like to live through the demands of a trial. They can often tell whether weekly clinic visits are realistic for an older patient who no longer drives, whether three additional biopsies might deter people from enrolling, or which eligibility rules could quietly exclude the people most likely to benefit. No single advocate speaks for every patient, and lived experience is not the same as representative data. It is a vantage point that a committee made up only of clinicians and scientists can easily miss.

But here is what often happens when a patient advocate raises one of these concerns at a Scientific Review Committee meeting: someone gently redirects the conversation. “That sounds like an ethics question. The Institutional Review Board will look at that.” The advocate’s concern gets handed off, and by the time it reaches the ethics review, the trial design is already locked in.

This article argues that this handoff is a mistake. Patient burden is not only an ethics question. It is also a scientific question, because trials that ask too much of patients fail to enroll enough people, lose participants partway through, and produce results that do not apply to the wider patient community. When committees treat burden as someone else’s problem, they miss the chance to fix the trial when it is still possible to fix.

I offer a name for this pattern: jurisdictional dismissal. I describe how it shows up in practice, why it weakens science, and what review committees can do about it. The fix is straightforward: treat patient burden as a scientific feasibility question, recognize advocates as scientific reviewers rather than ethics consultants, and weigh their concerns seriously, alongside concerns about statistical power or biological plausibility. The patient voice belongs in the room where the trial is designed, not only in the room where it is approved.

**Clinical trial number** Not applicable.

## Introduction

A scenario, drawn loosely from composite experience on a Scientific Review Committee:

A protocol arrives for a phase II trial in older adults with a common solid tumor. The advocate on the committee notes that the schedule requires twelve in-person clinic visits over six months, that two of the three required biopsies are not clinically indicated, and that the eligibility criteria exclude patients with stable, treated brain metastases. The advocate raises each concern in turn. The conversation that follows is polite. A senior member responds that the visit schedule is required by the pharmacokinetic sub-study. Another notes that the IRB will scrutinize the biopsy risks during ethics review. A third observes that the eligibility criteria are appropriate to manage participant safety, again something the IRB will consider. The protocol is approved with minor revisions. The trial subsequently struggles to enroll, closes early, and produces underpowered results that change no one’s practice.

Each individual response in that meeting was reasonable on its own terms. Together, they represent a pattern I will call jurisdictional dismissal: the systematic deflection of patient-centered feasibility concerns away from scientific review on the grounds that they belong somewhere else. The ‘somewhere else’ is almost always the Institutional Review Board (IRB). The implicit theory is that scientific review handles the science and ethics review handles the patient experience. That theory is wrong, and it is wrong in a way that costs the field trials, time, and credibility.

Patient and public involvement (PPI) in research has matured to the point where many institutions now seat trained advocates as full voting members of their Scientific Review Committees [1, 2]. The Stanford Cancer Institute model, on which I draw here, treats advocates as members of equal standing, with the same right to request modifications and to vote on approval as any clinical or scientific reviewer [3]. The premise of this arrangement is sound: by placing advocates upstream of the IRB, in the body that evaluates scientific design, institutions ensure that the patient perspective shapes the architecture of the trial rather than the wrapper around it [4]. Advocates were added to these committees for precisely this purpose: to bring the realities of the patient experience to bear while the design can still change. That is the work jurisdictional dismissal, described below, quietly prevents them from doing. The premise only delivers, however, if the committee is willing to treat the advocate’s contribution as scientific rather than as a permitted-but-peripheral ethical voice.

This Comment makes three claims. First, jurisdictional dismissal is a recognizable, recurring pattern that converts the structural advantage of upstream advocate placement into a procedural inconvenience. Second, it rests on a confusion between two distinct questions, only one of which the IRB is positioned to answer. Third, the fix is structural, not interpersonal. I close with four concrete changes to SRC practice.

## The two questions

Patient burden raises two related but separable questions that map onto two different review bodies.

The scientific feasibility question is: given the realistic constraints of the patient population this trial is designed to serve, can this protocol enroll enough participants and retain them long enough to generate valid, generalizable evidence? This question turns on accrual, retention, data completeness, and external validity. It is properly the SRC’s question because the SRC is the body charged with evaluating whether a study is well-designed to answer the research question it poses [5, 6]. A trial that cannot enroll a representative sample, or cannot retain enrolled participants through primary endpoint assessment, fails on scientific grounds before any ethical question arises.

The ethical acceptability question is: assuming this protocol can feasibly be executed, are the residual risks and burdens reasonable in relation to the anticipated benefits and the social value of the knowledge to be gained? This question turns on risk-benefit balance, equitable selection, and the adequacy of informed consent. It is properly the IRB’s question because the IRB is the body charged with protecting the rights and welfare of human research participants within the design that scientific review has approved [7, 8].

These questions are sequential and complementary, at least under the current regulatory arrangement, which assigns the evaluation of scientific design to the SRC and the protection of enrolled participants to the IRB. Some readers will reasonably regard that division as itself part of the problem, for reasons close to the ones I raise here; I take the arrangement as given and argue for fuller use of the stage it already provides. The IRB cannot answer the second question well if the SRC has not seriously engaged the first. If the protocol arrives at the IRB with a visit schedule that quietly guarantees attrition, the IRB is poorly positioned to redesign it. Its formal scope to request modifications may be broader than it is often treated in practice, but by that stage the design is effectively settled, and wholesale methodological redesign is rarely what ethics review undertakes. It can ask whether the burden is ethically acceptable for the participants who do enroll, but it cannot ask whether the trial as designed is scientifically capable of producing the evidence that would justify the burden in the first place. That question has already been answered, by default, in the SRC.

The line between the two questions is not always sharp. How acceptable a burden is judged to be can itself shape whether a trial is feasible. Oncology trials, for instance, are often demanding, with frequent visits, serial imaging, and repeated biopsies, yet many patients accept that burden in light of the potential benefit, and such trials accrue. The same objective burden may be readily tolerated in one population and prohibitive in another. Cases like these are best treated as both scientific and ethical at once, rather than assigned wholesale to either committee. That is not a reason to treat burden as only an ethical matter, which is the move this Comment resists.

Table 1 makes the distinction concrete across common categories of advocate concern.

**Table 1:** Distinguishing the scientific feasibility question (SRC) from the ethical acceptability question (IRB)

Concern raised by advocate	SRC question (scientific feasibility)	IRB question (ethical acceptability)	Address first at
Weekly visits for 52 weeks in a rural elderly population	Will this schedule prevent us from enrolling and retaining a representative sample, undermining statistical power and generalizability?	If participants do enroll, is the burden of weekly travel ethically acceptable given the anticipated benefit?	SRC
Three research-only biopsies that are not clinically indicated	Will the additional biopsy requirement deter enrollment or accelerate dropout, compromising data completeness?	Are research-only biopsies justified by the scientific value of the data they generate, and are risks adequately disclosed?	SRC
Eligibility excludes patients with treated, stable brain metastases	Does this exclusion produce a study population so unrepresentative that the results cannot be generalized to standard practice?	If the population is broadened, are the additional participants adequately protected?	SRC
A 27-page consent document dense with technical language	Will an inaccessible consent process suppress enrollment or yield consent that does not reflect genuine comprehension?	Does the document meet regulatory standards for informed, voluntary consent?	SRC
A study with significant toxicity and no direct therapeutic benefit	Is the risk-benefit profile likely to permit enrollment in a population that has alternatives?	Is enrollment ethically justifiable given the social value of the knowledge sought?	SRC
Procedures for minimizing and monitoring individual participant risk, such as safety stopping rules	Not primarily an SRC question; these safeguards do not determine whether the study can generate valid evidence.	Are foreseeable risks minimized and adequately monitored for those who enroll?	IRB (ethics review)
The specific wording and process for obtaining informed consent	Not an SRC question once the protocol can feasibly enroll and complete.	Does the consent process ensure informed, voluntary participation?	IRB (ethics review)

Every concern listed is ultimately reviewed by both bodies; the final column indicates where the concern should be raised and resolved first. The first five rows are hybrid scientific-ethical concerns that belong first at the SRC; the final two are concerns that arise only in ethics review

## How jurisdictional dismissal works

The dismissal rarely takes the form of an explicit refusal to engage. It usually comes wrapped in deference to a different oversight body or in appeals to an existing scientific rationale that is not itself in question.

The most common form is the IRB handoff. An advocate raises a concern about visit frequency or invasiveness, and a colleague responds, in good faith, that the IRB will weigh the burden during ethics review. The handoff treats the advocate’s concern as a question about whether the burden is acceptable rather than a question about whether the population the trial is meant to serve can realistically sustain it. The first is an ethics question. The second is a feasibility question. Only the second can be answered before the design is locked.

A second form is the appeal to scientific necessity. A protocol calls for serial sampling at intervals dictated by a pharmacokinetic sub-study; an advocate notes that the schedule will be punishing for fatigued patients on active treatment; the response is that the schedule is required by the science. Sometimes it is. Often the underlying scientific requirement is a particular density of measurement, not a particular schedule, and there is room to design around the patient. The appeal to necessity ends the conversation before that exploration begins.

A third form, subtler than the others, is the relegation of patient input to a category called “perspective” or “feedback,” distinguished from the “science” that the rest of the committee is doing. Advocates are thanked for their input and their input is recorded; the protocol moves forward without modification. The committee has heard from the patient voice. It has not been changed by it.

None of these moves is necessarily made in bad faith. Each can be the product of genuine confusion about which body owns which question, or of a sincere belief that protecting science from non-scientists is part of an SRC member’s duty. Practice also varies. Some SRCs, and some IRBs, do take these questions up directly, and what I describe is a strong tendency rather than a universal rule, though a tendency familiar to many who have served on these committees. The cumulative effect, however, is to neutralize the structural reform that putting advocates on the SRC was supposed to accomplish. If feasibility concerns are not the SRC’s business, then the only place they can be addressed is the IRB, and by then the design is settled. The patient advocate, formally seated at the upstream review, has been functionally relocated to the downstream one.

## Why it matters scientifically

The case for treating patient burden as a scientific question does not require any new conceptual move. It requires only that the field take seriously what it already knows about why trials fail.

Accrual failure is one of the most common reasons clinical trials close without producing usable evidence. Estimates vary by setting, but a substantial share of trials that open never reach their target enrollment, and a meaningful fraction close early on accrual grounds [9, 10]. Burden is not the only driver of accrual failure, but it is a recognized one, and it is among the few drivers that can be addressed by design. A protocol that requires participants to attend the clinic twice as often as standard care, or to undergo procedures they would not otherwise face, raises the practical threshold for taking part in ways that compound across populations facing transportation barriers, caregiving responsibilities, or financial precarity [11].

Retention is the same problem at a different point in the timeline. Participants who enroll under demanding protocols drop out at higher rates, and the dropouts are not random. They are concentrated among participants for whom the burden was always going to be hardest to absorb. The result is missing data that is missing not at random, with predictable threats to internal validity and predictable distortions of subgroup analysis. A protocol that loses 30% of its participants by the primary endpoint is not a protocol that has 70% of its evidence; it is a protocol whose remaining evidence comes from a self-selected subset that may not resemble the population the results will be applied to [12].

Generalizability is the third problem. Eligibility criteria that exclude common comorbidities, prior therapies, or clinical situations narrow the trial population to a slice that does not match the patients clinicians actually treat. The trial may produce a clean efficacy signal in the slice and leave the field unable to say whether the result applies to the whole [13]. Advocates are often the first to point this out, because they are in contact with patient communities whose members would be excluded by criteria that look reasonable on paper.

None of this implies that only advocates can raise these issues. Clinicians often have a good sense of what their patients will find difficult, and they sometimes underestimate how much patients are willing to take on. The argument here is not that feasibility concerns require a patient advocate, but that they require someone, at the stage where the design can still change, and that advocates are well placed to raise them and are often the first to do so. Their role is not only to object. An advocate close to a community may also argue for a study that clinicians assume is too burdensome to accrue, correcting a misjudgment in the other direction. The balance of evidence still favors patient and community involvement as the most reliable way to surface and act on these concerns. Patient engagement has been linked to research that better reflects patient priorities [14], and a systematic review and meta-analysis found that involving people with lived experience of the condition under study was associated with significantly higher trial enrolment [15]. Involvement may not be strictly necessary to ask the feasibility question, but on current evidence it remains the best-supported way to ask it well.

None of these failure modes is exotic. All are well documented in the methodological literature. None is properly the IRB’s to fix. They are scientific failures, and they happen at the design stage, which is the SRC’s stage.

## Four changes for scientific review committees

The fix is not to add a procedural step or to require additional advocate training. The fix is structural, and it can be implemented within existing committee architecture.

First, name feasibility as a scientific review criterion. SRC charters and review rubrics should explicitly list patient burden, accrual feasibility, and protocol acceptability for the target population among the scored dimensions of scientific review, alongside design, statistical plan, and rigor. The criterion should be framed as a feasibility question, not an ethics question: will this design enroll, retain, and produce generalizable results in the population it targets? Naming the criterion makes it harder to dismiss concerns raised against it.

Second, give committee chairs a default response to feasibility concerns that does not route them to the IRB. When an advocate raises a burden concern, the chair’s first move should be to ask the proposing investigator how the design accommodates the population’s realistic constraints, and what the team’s own accrual modeling assumes about the burden in question. Only after that question has been answered, on the merits, should anyone raise the question of whether residual burden is ethically acceptable. The IRB can have its conversation. The SRC needs to have its first.

Third, require a written response to advocate-flagged concerns before approval. This is a procedural mechanism that several institutions already use under labels like “Response Required.” The point is not to give advocates veto power, which they do not and should not have. The point is to create accountability: a concern that the committee accepted as legitimate produces a written investigator response that the committee evaluates before it votes. Concerns that survive the investigator’s response shape the protocol or shape the vote. They do not disappear into meeting minutes.

Fourth, train scientific reviewers, not only advocates. The literature on PPI training is dominated by programs that teach advocates the language of science. This is necessary and good. It is also incomplete. Scientific reviewers need parallel training in why patient burden is a scientific concern and how feasibility threats degrade the evidence their committees are charged with protecting. The training does not need to be elaborate. A single session built around concrete cases, including cases where dismissed feasibility concerns predicted accrual failures that subsequently happened, would shift the conversation in many committees.

These changes place the first responsibility with the SRC, where the design is still open. A complementary safeguard belongs at the IRB. The Common Rule already requires that every IRB include at least one member whose primary concerns are scientific [16], so ethics review is equipped to confirm that feasibility-relevant burden has been taken seriously rather than to accept the SRC’s judgment by default. In the pragmatic-trial literature especially, questions of accrual, statistical power, and enrollment patterns already surface in ethics and safety review. None of this displaces the SRC. The goal is met as long as some review body engages these questions on the merits, and the trial is best served when both do.

## Conclusion

Seating patient advocates on Scientific Review Committees was a structural reform. It was meant to put the patient perspective in the room where the scientific design of a trial is decided, not only in the room where its ethics are reviewed. The reform delivers when the committee accepts that what the advocate brings is scientific evidence about feasibility, grounded in experience of the patient community that other reviewers may not share. It does not deliver when the committee, by reflex or by category error, treats the advocate’s concerns as ethics issues to be handled later by someone else.

The fix does not require new bodies, new policies, or new resources. It requires that committees recognize a question they already have the authority to ask: whether the trial in front of them, as designed, can do what it claims it can do, in the patients it claims to serve. That is a scientific question. The patient advocate is often the member best placed to ask it. The committee’s job is to listen as if the answer matters to the science, because it does.

## Data Availability

No datasets were generated or analysed during the current study.

## References

[1] Perlmutter J, Bell SK, Darling MR. Patient advocates and researchers as partners in cancer research: a winning combination. Am Soc Clin Oncol Educ Book. 2013;33:e639–44. 10.1200/EDBK_100035 37167582

[2] Coordinating Center for Clinical Trials, National Cancer Institute. Patient advocate steering committee. https://www.cancer.gov/about-nci/organization/ccct/steering-committees/patient-advocate. Accessed 22 April 2026.

[3] Stanford Cancer Institute. Redefining cancer research with trained patient advocates. https://med.stanford.edu/cancer/about/news/patient-advocates.html. Accessed 22 April 2026.

[4] Stewart DJ, Whitney SN, Kurzrock R. Equipoise lost: ethics, costs, and the regulation of cancer clinical research. J Clin Oncol. 2010;28(17):2925–35. 20406924 10.1200/JCO.2009.27.5404

[5] Infonetica. Role of scientific review committees for quality assurance. Accessed 22 April 2026. https://www.infonetica.net/articles/Scientific-Review-Committee

[6] St. Jude Children’s Research Hospital. Clinical Trials Scientific Review Committee. https://www.stjude.org/research/why-st-jude/shared-resources/clinical-trials-scientific-review-committee.html. Accessed 22 April 2026.

[7] U.S. Food and Drug Administration. Institutional Review Boards Frequently Asked Questions. https://www.fda.gov/regulatory-information/search-fda-guidance-documents/institutional-review-boards-frequently-asked-questions. Accessed 22 April 2026.

[8] Grady C. Institutional review boards: purpose and challenges. Chest. 2015;148(5):1148–55. 26042632 10.1378/chest.15-0706 PMC4631034

[9] Carlisle B, Kimmelman J, Ramsay T, MacKinnon N. Unsuccessful trial accrual and human subjects protections: an empirical analysis of recently closed trials. Clin Trials. 2015;12(1):77–83. 25475878 10.1177/1740774514558307 PMC4516407

[10] Bennette CS, Ramsey SD, McDermott CL, Carlson JJ, Basu A, Veenstra DL. Predicting low accrual in the National Cancer Institute’s cooperative group clinical trials. J Natl Cancer Inst. 2016;108(2):djv324. 26714555 10.1093/jnci/djv324 PMC5964887

[11] Unger JM, Cook E, Tai E, Bleyer A. The role of clinical trial participation in cancer research: barriers, evidence, and strategies. Am Soc Clin Oncol Educ Book. 2016;35:185–98. 27249699 10.14694/EDBK_156686 PMC5495113

[12] Little RJ, D’Agostino R, Cohen ML, Dickersin K, Emerson SS, Farrar JT, et al. The prevention and treatment of missing data in clinical trials. N Engl J Med. 2012;367(14):1355–60. 23034025 10.1056/NEJMsr1203730 PMC3771340

[13] Kim ES, Bruinooge SS, Roberts S, Ison G, Lin NU, Gore L, et al. Broadening eligibility criteria to make clinical trials more representative: American Society of Clinical Oncology and Friends of Cancer Research joint research statement. J Clin Oncol. 2017;35(33):3737–44. 28968170 10.1200/JCO.2017.73.7916 PMC5692724

[14] Domecq JP, Prutsky G, Elraiyah T, Wang Z, Nabhan M, Shippee N, et al. Patient engagement in research: a systematic review. BMC Health Serv Res. 2014;14:89. 24568690 10.1186/1472-6963-14-89 PMC3938901

[15] Crocker JC, Ricci-Cabello I, Parker A, Hirst JA, Chant A, Petit-Zeman S, et al. Impact of patient and public involvement on enrolment and retention in clinical trials: systematic review and meta-analysis. BMJ. 2018;363:k4738. 30487232 10.1136/bmj.k4738 PMC6259046

[16] U.S. Department of Health and Human Services. Federal policy for the protection of human subjects (Common Rule). 45 CFR 46.107, composition of institutional review boards. Washington, DC: U.S. Department of Health and Human Services.

